# Supplementary material for: Perturbations of the T-cell immune repertoire in kidney transplant rejection
Source: Front Immunol. 2022 Nov 16;13:1012042. doi: 10.3389/fimmu.2022.1012042 (PMC9709472; doi:10.3389/fimmu.2022.1012042)
Supplement: Supplementary file 1 [file DataSheet_1.docx]

**Supplemental Figures**

**Supplemental Figure 1. V and J Gene Usage Among Baseline Samples**. Cumulative frequency of V [A] and J [B] genes among the 92 AR and 98 STA baseline samples. P values determined by wilcox rank sum test, * p<0.05, ** p<0.01.

**Supplemental Figure 2. Change in repertoire clonality is not awith acute rejection.** (A) Patients with acute rejection do not show a significantly different change in repertoire clonality relative to stable patients. (B) There is not significant difference in repertoire clonality in ABMR compared to non-ABMR samples.

**Supplemental Figure 3. Morisita Index correlates with post-transplant time until AR episode.** Among the AR cohort (ABMR, TCMR, and mixed AR included that had features of both TCMR and ABMR), Morisita Index was significantly correlated with time to AR with spearman’s Rho, R=0.33, P=0.029.

**Supplemental Figure 4. V and J Gene Usage Does Not Vary Post Transplant.** Cumulative frequency of V [A] and J [B] genes among the 49 AR and 49 STA Post-transplant samples. P values determined by wilcox rank sum test, * p<0.05.
